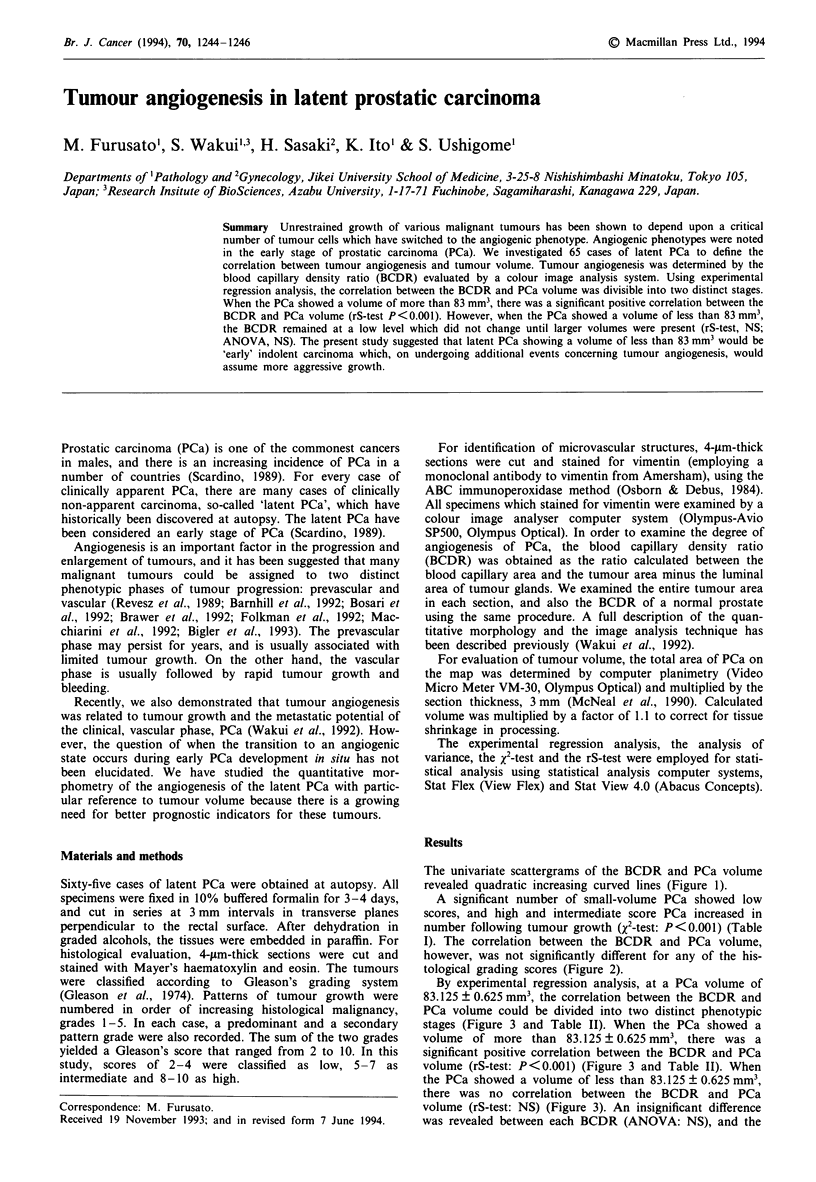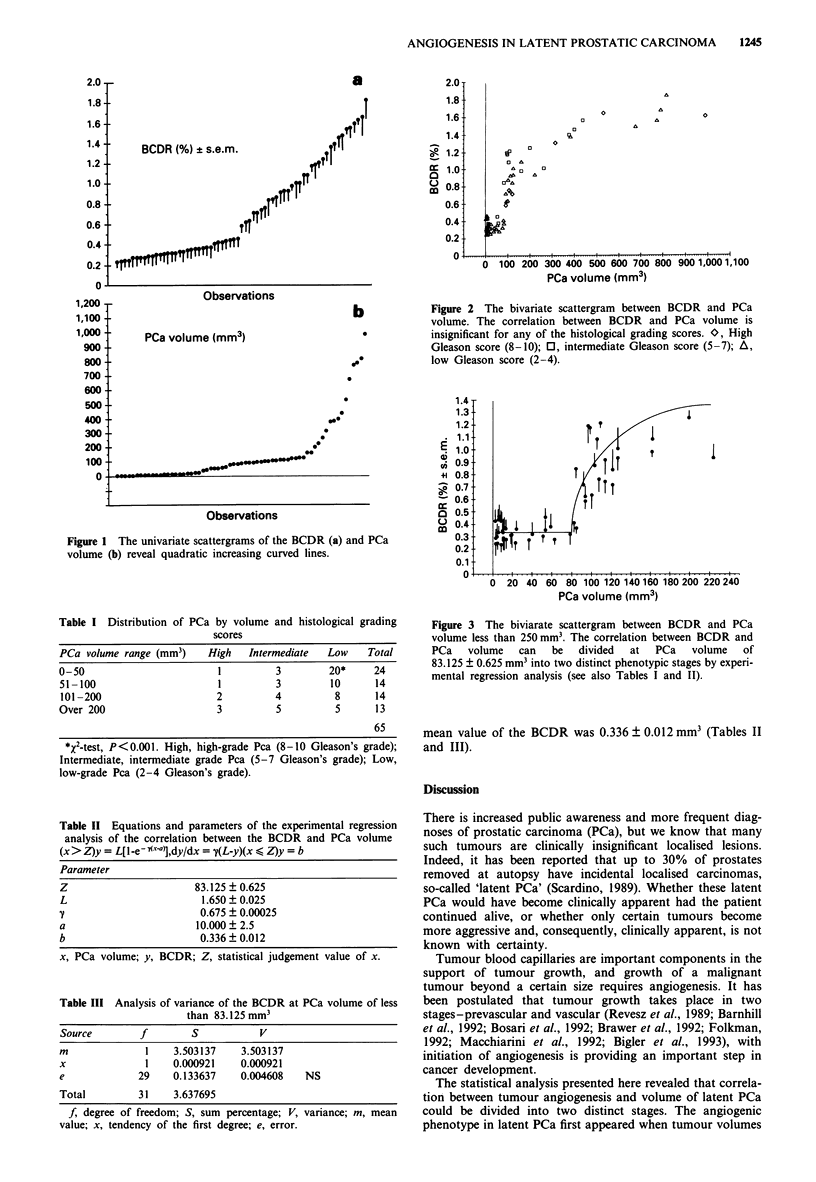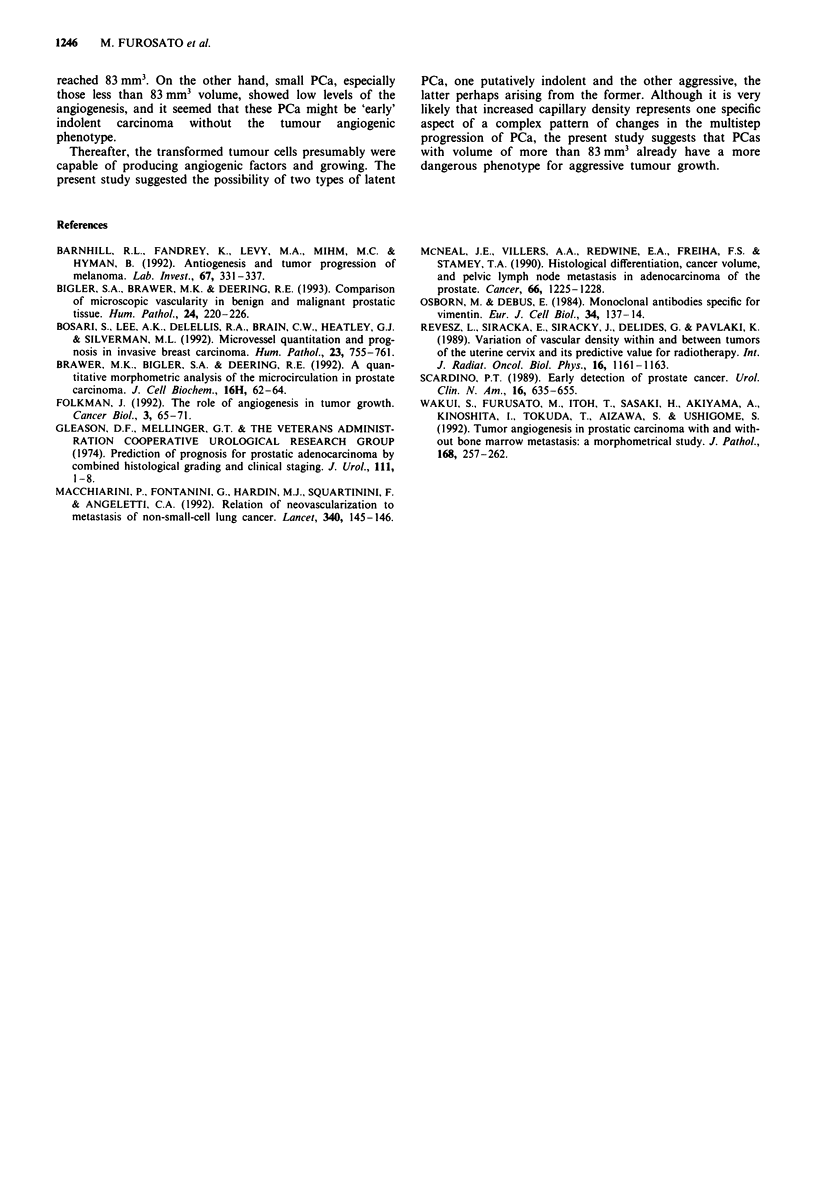# Tumour angiogenesis in latent prostatic carcinoma.

**DOI:** 10.1038/bjc.1994.480

**Published:** 1994-12

**Authors:** M. Furusato, S. Wakui, H. Sasaki, K. Ito, S. Ushigome

**Affiliations:** Department of Pathology, Jikei University School of Medicine, Tokyo, Japan.

## Abstract

Unrestrained growth of various malignant tumours has been shown to depend upon a critical number of tumour cells which have switched to the angiogenic phenotype. Angiogenic phenotypes were noted in the early stage of prostatic carcinoma (PCa). We investigated 65 cases of latent PCa to define the correlation between tumour angiogenesis and tumour volume. Tumour angiogenesis was determined by the blood capillary density ratio (BCDR) evaluated by a colour image analysis system. Using experimental regression analysis, the correlation between the BCDR and PCa volume was divisible into two distinct stages. When the PCa showed a volume of more than 83 mm3, there was a significant positive correlation between the BCDR and PCa volume (rS-test P < 0.001). However, when the PCa showed a volume of less than 83 mm3, the BCDR remained at a low level which did not change until larger volumes were present (rS-test, NS; ANOVA, NS). The present study suggested that latent PCa showing a volume of less than 83 mm3 would be 'early' indolent carcinoma which, on undergoing additional events concerning tumour angiogenesis, would assume more aggressive growth.


 © Macmillan Press Ltd., 1994

### Tumour angiogenesis in latent prostatic carcinoma

M. Furusato[1], S. Wakui[1,3], H. Sasaki[2], K. Ito[1] & S. Ushigome[1]

Departments of [1]Pathology and [2]Gynecology, Jikei University School of Medicine, 3-25-8 Nishishimbashi Minatoku, Tokyo 105, Japan; [3]Research Insitute of BioSciences, Azabu University, 1-17-71 Fuchinobe, Sagamiharashi, Kanagawa 229, Japan.

**Summary** Unrestrained growth of various malignant tumours has been shown to depend upon a critical number of tumour cells which have switched to the angiogenic phenotype. Angiogenic phenotypes were noted in the early stage of prostatic carcinoma (PCa). We investigated 65 cases of latent PCa to define the correlation between tumour angiogenesis and tumour volume. Tumour angiogenesis was determined by the blood capillary density ratio (BCDR) evaluated by a colour image analysis system. Using experimental regression analysis, the correlation between the BCDR and PCa volume was divisible into two distinct stages. When the PCa showed a volume of more than 83 $mm^3$, there was a significant positive correlation between the BCDR and PCa volume (rS-test $P<0.001$). However, when the PCa showed a volume of less than 83 $mm^3$, the BCDR remained at a low level which did not change until larger volumes were present (rS-test, NS; ANOVA, NS). The present study suggested that latent PCa showing a volume of less than 83 $mm^3$ would be 'early' indolent carcinoma which, on undergoing additional events concerning tumour angiogenesis, would assume more aggressive growth.

Prostatic carcinoma (PCa) is one of the commonest cancers in males, and there is an increasing incidence of PCa in a number of countries (Scardino, 1989). For every case of clinically apparent PCa, there are many cases of clinically non-apparent carcinoma, so-called 'latent PCa', which have historically been discovered at autopsy. The latent PCa have been considered an early stage of PCa (Scardino, 1989).

Angiogenesis is an important factor in the progression and enlargement of tumours, and it has been suggested that many malignant tumours could be assigned to two distinct phenotypic phases of tumour progression: prevascular and vascular (Revesz *et al.*, 1989; Barnhill *et al.*, 1992; Bosari *et al.*, 1992; Brawer *et al.*, 1992; Folkman *et al.*, 1992; Macchiarini *et al.*, 1992; Bigler *et al.*, 1993). The prevascular phase may persist for years, and is usually associated with limited tumour growth. On the other hand, the vascular phase is usually followed by rapid tumour growth and bleeding.

Recently, we also demonstrated that tumour angiogenesis was related to tumour growth and the metastatic potential of the clinical, vascular phase, PCa (Wakui *et al.*, 1992). However, the question of when the transition to an angiogenic state occurs during early PCa development *in situ* has not been elucidated. We have studied the quantitative morphometry of the angiogenesis of the latent PCa with particular reference to tumour volume because there is a growing need for better prognostic indicators for these tumours.

### Materials and methods

Sixty-five cases of latent PCa were obtained at autopsy. All specimens were fixed in 10% buffered formalin for 3-4 days, and cut in series at 3 mm intervals in transverse planes perpendicular to the rectal surface. After dehydration in graded alcohols, the tissues were embedded in paraffin. For histological evaluation, 4-μm-thick sections were cut and stained with Mayer's haematoxylin and eosin. The tumours were classified according to Gleason's grading system (Gleason *et al.*, 1974). Patterns of tumour growth were numbered in order of increasing histological malignancy, grades 1-5. In each case, a predominant and a secondary pattern grade were also recorded. The sum of the two grades yielded a Gleason's score that ranged from 2 to 10. In this study, scores of 2-4 were classified as low, 5-7 as intermediate and 8-10 as high.

Correspondence: M. Furusato.
Received 19 November 1993; and in revised form 7 June 1994.

For identification of microvascular structures, 4-μm-thick sections were cut and stained for vimentin (employing a monoclonal antibody to vimentin from Amersham), using the ABC immunoperoxidase method (Osborn & Debus, 1984). All specimens which stained for vimentin were examined by a colour image analyser computer system (Olympus-Avio SP500, Olympus Optical). In order to examine the degree of angiogenesis of PCa, the blood capillary density ratio (BCDR) was obtained as the ratio calculated between the blood capillary area and the tumour area minus the luminal area of tumour glands. We examined the entire tumour area in each section, and also the BCDR of a normal prostate using the same procedure. A full description of the quantitative morphology and the image analysis technique has been described previously (Wakui *et al.*, 1992).

For evaluation of tumour volume, the total area of PCa on the map was determined by computer planimetry (Video Micro Meter VM-30, Olympus Optical) and multiplied by the section thickness, 3 mm (McNeal *et al.*, 1990). Calculated volume was multiplied by a factor of 1.1 to correct for tissue shrinkage in processing.

The experimental regression analysis, the analysis of variance, the $\chi^2$-test and the rS-test were employed for statistical analysis using statistical analysis computer systems, Stat Flex (View Flex) and Stat View 4.0 (Abacus Concepts).

### Results

The univariate scattergrams of the BCDR and PCa volume revealed quadratic increasing curved lines (Figure 1).

A significant number of small-volume PCa showed low scores, and high and intermediate score PCa increased in number following tumour growth ($\chi^2$-test: $P<0.001$) (Table I). The correlation between the BCDR and PCa volume, however, was not significantly different for any of the histological grading scores (Figure 2).

By experimental regression analysis, at a PCa volume of $83.125 \pm 0.625$ $mm^3$, the correlation between the BCDR and PCa volume could be divided into two distinct phenotypic stages (Figure 3 and Table II). When the PCa showed a volume of more than $83.125 \pm 0.625$ $mm^3$, there was a significant positive correlation between the BCDR and PCa volume (rS-test: $P<0.001$) (Figure 3 and Table II). When the PCa showed a volume of less than $83.125 \pm 0.625$ $mm^3$, there was no correlation between the BCDR and PCa volume (rS-test: NS) (Figure 3). An insignificant difference was revealed between each BCDR (ANOVA: NS), and the

**Figure 1** The univariate scattergrams of the BCDR (**a**) and PCa volume (**b**) reveal quadratic increasing curved lines.

**Table I** Distribution of PCa by volume and histological grading scores

| *PCa volume range* (mm³) | *High* | *Intermediate* | *Low* | *Total* |
|---|---|---|---|---|
| 0–50 | 1 | 3 | 20* | 24 |
| 51–100 | 1 | 3 | 10 | 14 |
| 101–200 | 2 | 4 | 8 | 14 |
| Over 200 | 3 | 5 | 5 | 13 |
| | | | | 65 |

*$\chi^2$-test, $P<0.001$. High, high-grade Pca (8–10 Gleason's grade); Intermediate, intermediate grade Pca (5–7 Gleason's grade); Low, low-grade Pca (2–4 Gleason's grade).

**Table II** Equations and parameters of the experimental regression analysis of the correlation between the BCDR and PCa volume $(x>Z) y = L[1\text{-}e^{-\gamma(x-a)}], dy/dx = \gamma(L-y)(x \leqslant Z) y = b$

| *Parameter* | |
|---|---|
| $Z$ | 83.125 ± 0.625 |
| $L$ | 1.650 ± 0.025 |
| $\gamma$ | 0.675 ± 0.00025 |
| $a$ | 10.000 ± 2.5 |
| $b$ | 0.336 ± 0.012 |

$x$, PCa volume; $y$, BCDR; $Z$, statistical judgement value of $x$.

**Table III** Analysis of variance of the BCDR at PCa volume of less than 83.125 mm³

| *Source* | *f* | *S* | *V* | |
|---|---|---|---|---|
| $m$ | 1 | 3.503137 | 3.503137 | |
| $x$ | 1 | 0.000921 | 0.000921 | |
| $e$ | 29 | 0.133637 | 0.004608 | NS |
| Total | 31 | 3.637695 | | |

$f$, degree of freedom; $S$, sum percentage; $V$, variance; $m$, mean value; $x$, tendency of the first degree; $e$, error.

**Figure 2** The bivariate scattergram between BCDR and PCa volume. The correlation between BCDR and PCa volume is insignificant for any of the histological grading scores. ◇, High Gleason score (8–10); □, intermediate Gleason score (5–7); △, low Gleason score (2–4).

**Figure 3** The biviarate scattergram between BCDR and PCa volume less than 250 mm³. The correlation between BCDR and PCa volume can be divided at PCa volume of 83.125 ± 0.625 mm³ into two distinct phenotypic stages by experimental regression analysis (see also Tables I and II).

mean value of the BCDR was 0.336 ± 0.012 mm³ (Tables II and III).

### Discussion

There is increased public awareness and more frequent diagnoses of prostatic carcinoma (PCa), but we know that many such tumours are clinically insignificant localised lesions. Indeed, it has been reported that up to 30% of prostates removed at autopsy have incidental localised carcinomas, so-called 'latent PCa' (Scardino, 1989). Whether these latent PCa would have become clinically apparent had the patient continued alive, or whether only certain tumours become more aggressive and, consequently, clinically apparent, is not known with certainty.

Tumour blood capillaries are important components in the support of tumour growth, and growth of a malignant tumour beyond a certain size requires angiogenesis. It has been postulated that tumour growth takes place in two stages–prevascular and vascular (Revesz *et al.*, 1989; Barnhill *et al.*, 1992; Bosari *et al.*, 1992; Brawer *et al.*, 1992; Folkman, 1992; Macchiarini *et al.*, 1992; Bigler *et al.*, 1993), with initiation of angiogenesis is providing an important step in cancer development.

The statistical analysis presented here revealed that correlation between tumour angiogenesis and volume of latent PCa could be divided into two distinct stages. The angiogenic phenotype in latent PCa first appeared when tumour volumes reached 83 $mm^3$. On the other hand, small PCa, especially those less than 83 $mm^3$ volume, showed low levels of the angiogenesis, and it seemed that these PCa might be 'early' indolent carcinoma without the tumour angiogenic phenotype.

Thereafter, the transformed tumour cells presumably were capable of producing angiogenic factors and growing. The present study suggested the possibility of two types of latent PCa, one putatively indolent and the other aggressive, the latter perhaps arising from the former. Although it is very likely that increased capillary density represents one specific aspect of a complex pattern of changes in the multistep progression of PCa, the present study suggests that PCas with volume of more than 83 $mm^3$ already have a more dangerous phenotype for aggressive tumour growth.

### References

BARNHILL, R.L., FANDREY, K., LEVY, M.A., MIHM, M.C. & HYMAN, B. (1992). Antiogenesis and tumor progression of melanoma. *Lab. Invest.*, **67**, 331-337.

BIGLER, S.A., BRAWER, M.K. & DEERING, R.E. (1993). Comparison of microscopic vascularity in benign and malignant prostatic tissue. *Hum. Pathol.*, **24**, 220-226.

BOSARI, S., LEE, A.K., DELELLIS, R.A., BRAIN, C.W., HEATLEY, G.J. & SILVERMAN, M.L. (1992). Microvessel quantitation and prognosis in invasive breast carcinoma. *Hum. Pathol.*, **23**, 755-761.

BRAWER, M.K., BIGLER, S.A. & DEERING, R.E. (1992). A quantitative morphometric analysis of the microcirculation in prostate carcinoma. *J. Cell Biochem.*, **16H**, 62-64.

FOLKMAN, J. (1992). The role of angiogenesis in tumor growth. *Cancer Biol.*, **3**, 65-71.

GLEASON, D.F., MELLINGER, G.T. & THE VETERANS ADMINISTRATION COOPERATIVE UROLOGICAL RESEARCH GROUP (1974). Prediction of prognosis for prostatic adenocarcinoma by combined histological grading and clinical staging. *J. Urol.*, **111**, 1-8.

MACCHIARINI, P., FONTANINI, G., HARDIN, M.J., SQUARTININI, F. & ANGELETTI, C.A. (1992). Relation of neovascularization to metastasis of non-small-cell lung cancer. *Lancet*, **340**, 145-146.

MCNEAL, J.E., VILLERS, A.A., REDWINE, E.A., FREIHA, F.S. & STAMEY, T.A. (1990). Histological differentiation, cancer volume, and pelvic lymph node metastasis in adenocarcinoma of the prostate. *Cancer*, **66**, 1225-1228.

OSBORN, M. & DEBUS, E. (1984). Monoclonal antibodies specific for vimentin. *Eur. J. Cell Biol.*, **34**, 137-14.

REVESZ, L., SIRACKA, E., SIRACKY, J., DELIDES, G. & PAVLAKI, K. (1989). Variation of vascular density within and between tumors of the uterine cervix and its predictive value for radiotherapy. *Int. J. Radiat. Oncol. Biol. Phys.*, **16**, 1161-1163.

SCARDINO, P.T. (1989). Early detection of prostate cancer. *Urol. Clin. N. Am.*, **16**, 635-655.

WAKUI, S., FURUSATO, M., ITOH, T., SASAKI, H., AKIYAMA, A., KINOSHITA, I., TOKUDA, T., AIZAWA, S. & USHIGOME, S. (1992). Tumor angiogenesis in prostatic carcinoma with and without bone marrow metastasis: a morphometrical study. *J. Pathol.*, **168**, 257-262.